# Draft genome sequences of 53 *Lactococcus* and *Leuconostoc* strains isolated from two undefined DL-starter cultures

**DOI:** 10.1128/mra.00228-24

**Published:** 2024-05-24

**Authors:** Wenwen Li, Axel Soto-Serrano, Farhad M. Panah, Göksen Arik, Paulina Deptula, Daniella Lucena, Finn K. Vogensen, Lukasz Krych

**Affiliations:** 1 Section of Food Microbiology, Gut Health, and Fermentation, Department of Food Science, University of Copenhagen, Frederiksberg, Denmark; 2 Arla Innovation Centre, Arla Foods amba, Aarhus, Denmark; The University of Arizona, Tucson, Arizona, USA

**Keywords:** cheese, DL-starter culture, whole-genome sequencing, *Lactococcus*, *Leuconostoc*

## Abstract

This study presents the complete genomes of 53 strains of *Lactococcus* and *Leuconostoc* isolated from two undefined DL-starter cultures originating from Denmark, Tistrup, and P. The genomes were reconstructed using long-read, nanopore-based DNA sequencing, delivering comprehensive data set for comparative genomics and taxonomic classification, with potential utility in dairy fermentation processes.

## ANNOUNCEMENT

Back-slopped starter cultures for cheese production are typically undefined and vary between different cheeses (1, 2). Genomic characterization of undefined back-slopped starter cultures reveals the genetic basis of bacterial phenotypes and starter cultures properties (3).

This study analyzed 400 isolates, with 200 each from Tistrup and P starter cultures, named T and P isolates, respectively. *Lactococcus* strains were isolated using milk-based (skimmed milk with 0.2% tryptone, 50 mM MOPS from Sigma, and 0.003% Bromocresol purple from Darmstadt, Germany) and LM17 (M17 supplemented with 0.5% lactose) agar media. *Leuconostoc* strains were isolated from MRS agar media supplemented with 50 µg/mL Vancomycin (4–6). The isolated strains were purified three times on their respective agar media and preserved in 15% glycerol (vol/vol) at −60°C. Most reagents were sourced from HiMedia, Mumbai, India. All 400 isolates were sequenced using Oxford Nanopore Technology (ONT) to reconstruct circular contigs and identify species taxonomically. Average nucleotide identity (ANI) analyzed the 400 genomes for high-resolution similarity (7), identifying 53 distinct strains (Table 1).

**TABLE 1:** Genome features and accession numbers of the *Lactococcus* and *Leuconostoc* strains isolated from Tistrup and P starter cultures

										BUSCO analysis							
Isolate	Isolation site (source)	Taxonomy (species)	GenBank accession no.	SRA accession no.	Assembly size (bp)	No. of raw bases (bp)	No. of reads	*N*_50_ reads	G + C content (%)	Coverage (×)	No. of contigs	*N*_50_ (bp)	% of complete	% of complete and single copy	% of complete and duplicated	% of fragmented	% of missing
T1C9	Denmark (Tistrup starter culture)	*Lactococcus cremoris*	JAZHJH000000000	SRR27860930	2,581,963	277,056,382	65,910	5,656	35.78	107	10	2,379,820	96.8%	96.0%	0.8%	2.4%	0.8%
T2B1	Denmark (Tistrup starter culture)	*Lactococcus cremoris*	JAZHJI000000000	SRR27860929	2,588,282	305,614,895	71,539	5,780	35.76	118	11	2,379,805	97.6%	96.8%	0.8%	2.4%	0.0%
T1B4	Denmark (Tistrup starter culture)	*Lactococcus cremoris*	JAZHJJ000000000	SRR27860918	2,533,600	979,967,884	132,662	9,703	35.77	387	11	2,379,810	97.6%	96.8%	0.8%	2.4%	0.0%
T1A3	Denmark (Tistrup starter culture)	*Lactococcus cremoris*	JAZHJK000000000	SRR27860907	2,612,348	1,440,716,023	172,741	12,398	35.80	552	7	2,379,596	97.6%	96.8%	0.8%	2.4%	0.0%
T1B8	Denmark (Tistrup starter culture)	*Lactococcus cremoris*	JAZHJL000000000	SRR27860896	2,584,424	901,550,025	129,359	9,832	35.74	349	12	2,379,802	96.8%	96.0%	0.8%	2.4%	0.8%
T1A9	Denmark (Tistrup starter culture)	*Lactococcus cremoris*	JAZHJM000000000	SRR27860885	2,558,589	1,150,109,792	132,836	12,836	35.78	449	12	2,379,826	97.6%	96.8%	0.8%	2.4%	0.0%
T1D2	Denmark (Tistrup starter culture)	*Lactococcus cremoris*	JAZHJN000000000	SRR27860880	2,581,491	311,661,269	70,136	6,252	35.74	121	9	2,379,778	98.4%	97.6%	0.8%	1.6%	0.0%
T1G8	Denmark (Tistrup starter culture)	*Lactococcus cremoris*	JAZHJO000000000	SRR27860879	2,536,107	668,685,949	93,321	9,982	35.73	264	10	2,380,162	98.4%	97.6%	0.8%	1.6%	0.0%
T1D5	Denmark (Tistrup starter culture)	*Lactococcus cremoris*	JAZHJP000000000	SRR27860878	2,676,485	205,257,611	45,793	6,435	35.72	77	13	2,379,805	96.8%	96.0%	0.8%	2.4%	0.8%
T2F5	Denmark (Tistrup starter culture)	*Lactococcus cremoris*	JAZHJQ000000000	SRR27860877	2,667,581	369,638,548	58,936	8,962	35.66	139	18	2,379,810	97.6%	96.8%	0.8%	2.4%	0.0%
T1E11	Denmark (Tistrup starter culture)	*Lactococcus cremoris*	JAZHJR000000000	SRR27860928	2,613,099	483,642,746	125,228	4,971	35.79	185	12	2,381,264	97.6%	96.8%	0.8%	1.6%	0.8%
T1C6	Denmark (Tistrup starter culture)	*Lactococcus cremoris*	JAZHJS000000000	SRR27860927	2,611,951	358,543,452	84,129	5,858	35.77	137	12	2,381,959	97.6%	96.8%	0.8%	2.4%	0.0%
T1C12	Denmark (Tistrup starter culture)	*Lactococcus cremoris*	JAZHJT000000000	SRR27860926	2,628,357	984,020,776	147,618	9,808	35.78	374	10	2,379,808	97.6%	96.8%	0.8%	2.4%	0.0%
T1F3^*a*^	Denmark (Tistrup starter culture)	*Lactococcus cremoris*	JAZHJU000000000	SRR27860925	2,656,554	249,723,936	27,341	13,291	35.77	94	11	2,380,861	96.8%	96.0%	0.8%	3.2%	0.0%
T2G1	Denmark (Tistrup starter culture)	*Lactococcus cremoris*	JAZHJV000000000	SRR27860924	2,659,567	929,409,678	115,554	12,222	35.72	349	9	2,379,800	97.6%	96.8%	0.8%	2.4%	0.0%
P2F5	Denmark (*P* starter culture)	*Lactococcus cremoris*	JAZHJW000000000	SRR27860923	2,807,514	150,009,837	29,379	7,127	35.68	53	19	2,426,634	98.4%	97.6%	0.8%	1.6%	0.0%
P2G1^*b*^	Denmark (P starter culture)	*Lactococcus cremoris*	JAZHJX000000000	SRR27860922	2,547,717	1,281,656,366	121,134	15,000	35.88	503	5	2,097,027	97.6%	96.8%	0.8%	2.4%	0.0%
P2F12^*b*^	Denmark (P starter culture)	*Lactococcus cremoris*	JAZHJY000000000	SRR27860921	2,680,781	755,965,280	106,214	10,888	35.80	282	14	2,424,061	98.4%	97.6%	0.8%	1.6%	0.0%
P2A4^*a*^	Denmark (P starter culture)	*Lactococcus cremoris*	JAZHJZ000000000	SRR27860920	2,650,280	249,562,178	29,345	13,979	35.83	94	12	2,415,249	98.4%	97.6%	0.8%	1.6%	0.0%
P1G8	Denmark (P starter culture)	*Lactococcus cremoris*	JAZHKA000000000	SRR27860919	2,637,826	144,947,907	23,284	9,099	35.81	55	11	2,401,583	96.0%	95.2%	0.8%	3.2%	0.8%
T1F9	Denmark (Tistrup starter culture)	*Lactococcus cremoris*	JAZHKB000000000	SRR27860917	2,579,010	799,647,501	129,252	9,038	35.79	310	12	2,412,082	97.6%	96.8%	0.8%	1.6%	0.8%
P1F9	Denmark (P starter culture)	*Lactococcus cremoris*	JAZHKC000000000	SRR27860916	2,713,617	496,416,715	62,428	12,149	35.77	183	12	2,506,514	96.8%	96.0%	0.8%	3.2%	0.0%
P1E1	Denmark (P starter culture)	*Lactococcus cremoris*	JAZHKD000000000	SRR27860915	2,519,495	116,914,930	17,954	9,290	35.70	46	12	2,269,098	97.6%	97.6%	0.0%	2.4%	0.0%
P1E5	Denmark (P starter culture)	*Lactococcus cremoris*	JAZHKE000000000	SRR27860914	2,692,378	438,043,589	57,130	11,916	35.72	163	13	2,409,049	96.0%	95.2%	0.8%	4.0%	0.0%
P1E11	Denmark (P starter culture)	*Lactococcus cremoris*	JAZHKF000000000	SRR27860913	2,588,959	271,499,656	37,583	10,641	35.64	105	14	2,351,923	98.4%	97.6%	0.8%	1.6%	0.0%
P1C8	Denmark (P starter culture)	*Lactococcus cremoris*	JAZHKG000000000	SRR27860912	2,693,624	160,248,328	24,911	9,714	35.65	59	8	2,509,951	98.4%	98.4%	0.0%	1.6%	0.0%
P1F6	Denmark (P starter culture)	*Lactococcus cremoris*	JAZHKH000000000	SRR27860911	2,714,807	216,117,791	31,890	10,050	35.65	80	7	2,509,269	98.4%	98.4%	0.0%	1.6%	0.0%
P1B5	Denmark (P starter culture)	*Lactococcus cremoris*	JAZHKI000000000	SRR27860910	2,700,189	218,733,922	36,778	8,542	35.65	81	8	2,509,952	98.4%	98.4%	0.0%	1.6%	0.0%
P2E3	Denmark (P starter culture)	Lactococcus cremoris	JAZHKJ000000000	SRR27860909	2,693,463	765,900,986	121,223	9,196	35.65	284	7	2,509,950	98.4%	98.4%	0.0%	1.6%	0.0%
P1D9	Denmark (*P* starter culture)	Lactococcus cremoris	JAZHKK000000000	SRR27860908	2,703,842	325,416,035	50,257	9,660	35.65	120	7	2,511,279	98.4%	98.4%	0.0%	1.6%	0.0%
P2E5	Denmark (*P* starter culture)	Lactococcus cremoris	JAZHKL000000000	SRR27860906	2,719,525	930,660,162	139,335	9,825	35.63	342	8	2,509,961	98.4%	98.4%	0.0%	1.6%	0.0%
P2B7	Denmark (*P* starter culture)	Lactococcus cremoris	JAZHKM000000000	SRR27860905	2,692,455	740,572,076	105,199	10,347	35.66	275	6	2,509,950	98.4%	98.4%	0.0%	1.6%	0.0%
P1E10	Denmark (*P* starter culture)	Lactococcus cremoris	JAZHKN000000000	SRR27860904	2,744,887	95,584,044	14,306	10,080	35.62	35	8	2,501,251	98.4%	98.4%	0.0%	1.6%	0.0%
P1B8	Denmark (*P* starter culture)	Lactococcus cremoris	JAZHKO000000000	SRR27860903	2,693,185	103,127,727	16,256	9,425	35.65	38	8	2,509,946	98.4%	98.4%	0.0%	1.6%	0.0%
P2D7	Denmark (*P* starter culture)	Lactococcus cremoris	JAZHKP000000000	SRR27860902	2,726,444	436,431,928	66,235	9,812	35.63	160	8	2,509,955	98.4%	98.4%	0.0%	1.6%	0.0%
P2F11	Denmark (*P* starter culture)	Lactococcus cremoris	JAZHKQ000000000	SRR27860901	2,750,511	95,111,538	16,003	8,592	35.61	35	7	2,509,949	98.4%	98.4%	0.0%	1.6%	0.0%
P1A5	Denmark (*P* starter culture)	Lactococcus cremoris	JAZHKR000000000	SRR27860900	2,728,499	204,971,373	33,998	8,624	35.63	75	7	2,509,950	97.6%	97.6%	0.0%	2.4%	0.0%
P2D6	Denmark (*P* starter culture)	Lactococcus lactis	JAZHKS000000000	SRR27860899	2,657,212	571,184,472	112,107	7,007	35.19	215	14	2,412,586	98.4%	97.6%	0.8%	1.6%	0.0%
P2C1	Denmark (*P* starter culture)	Lactococcus lactis	JAZHKT000000000	SRR27860898	2,529,427	459,693,581	57,283	12,619	35.22	182	15	2,410,489	97.6%	96.8%	0.8%	2.4%	0.0%
P2E4	Denmark (*P* starter culture)	Lactococcus lactis	JAZHKU000000000	SRR27860897	2,625,794	586,754,092	107,740	7,740	35.16	223	12	2,413,455	98.4%	97.6%	0.8%	1.6%	0.0%
P2D5	Denmark (*P* starter culture)	Lactococcus lactis	JAZHKV000000000	SRR27860895	2,664,776	481,795,938	69,572	9,615	35.13	181	10	2,408,586	98.4%	97.6%	0.8%	1.6%	0.0%
T2A8	Denmark (Tistrup starter culture)	Lactococcus laudensis	JAZHKW000000000	SRR27860894	2,398,319	372,023,913	84,637	6,408	38.54	155	4	2,283,470	98.4%	97.6%	0.8%	1.6%	0.0%
T2G3	Denmark (Tistrup starter culture)	Lactococcus laudensis	JAZHKX000000000	SRR27860893	2,425,899	344,772,879	62,520	8,075	38.57	142	4	2,285,418	99.2%	98.4%	0.8%	0.8%	0.0%
T2H3	Denmark (Tistrup starter culture)	Lactococcus laudensis	JAZHKY000000000	SRR27860892	2,408,234	442,202,651	72,665	8,492	38.56	184	6	2,294,319	98.4%	97.6%	0.8%	1.6%	0.0%
T2D6^*b*^	Denmark (Tistrup starter culture)	Lactococcus laudensis	JAZHKZ000000000	SRR27860891	2,392,579	1,043,260,226	170,335	8,333	38.54	436	3	2,329,332	99.2%	98.4%	0.8%	0.8%	0.0%
T2C5	Denmark (Tistrup starter culture)	Lactococcus laudensis	JAZHLA000000000	SRR27860889	2,390,615	346,780,226	71,077	7,057	38.53	145	6	2,179,796	99.2%	98.4%	0.8%	0.8%	0.0%
T2H4	Denmark (Tistrup starter culture)	Lactococcus laudensis	JAZHLB000000000	SRR27860888	2,412,664	191,607,363	32,331	8,747	38.53	79	6	2,317,245	98.4%	97.6%	0.8%	1.6%	0.0%
T3A7	Denmark (Tistrup starter culture)	Leuconostoc mesenteroides	JAZHLC000000000	SRR27860887	1,763,109	463,764,209	81,379	8,381	38.39	263	3	1,714,386	96.8%	96.8%	0.0%	2.4%	0.8%
P3A4	Denmark (*P* starter culture)	Leuconostoc mesenteroides	JAZHLD000000000	SRR27860886	1,785,618	156,932,826	25,894	8,732	38.36	88	4	1,710,607	96.8%	96.8%	0.0%	2.4%	0.8%
T3A10	Denmark (Tistrup starter culture)	Leuconostoc mesenteroides	JAZHLE000000000	SRR27860884	1,745,760	763,126,018	150,100	7,142	38.46	437	6	1,677,559	96.0%	96.0%	0.0%	2.4%	1.6%
P3A12	Denmark (*P* starter culture)	Leuconostoc mesenteroides	JAZHLF000000000	SRR27860883	1,771,010	159,245,422	24,145	9,848	38.37	90	5	1,707,889	96.8%	96.0%	0.8%	2.4%	0.8%
P3A3^*b*^	Denmark (*P* starter culture)	Leuconostoc falkenbergense	JAZHLG000000000	SRR27860882	2,112,160	512,674,235	72,828	10,105	39.12	243	7	1,993,813	96.0%	96.0%	0.0%	3.2%	0.8%
P3A1	Denmark (*P* starter culture)	Leuconostoc falkenbergense	JAZHLH000000000	SRR27860881	2,126,919	100,359,973	22,114	6,263	39.11	47	8	2,028,352	96.0%	96.0%	0.0%	3.2%	0.8%

^*a*^ T1F3 and P2A4 were assembly with 60× coverage, the rest of the samples were assembled using all of the reads.

^*b*^ The Libraries for P2G1, P2F12, T2D6, and P3A3 were prepared using the Native Barcoding Kit, while the remaining samples were prepared with the Rapid Barcoding Kit.

Cryopreserved *Lactococcus* spp. and *Leuconostoc* spp. isolates were cultured on LM17 and MRS agar media, respectively, and incubated anaerobically at 30°C for 48 h. Single colonies were then inoculated into corresponding liquid medium and incubated under the same conditions (8). After incubation, bacterial cells were centrifuged at 5,000 × *g* for 10 min and washed with 1 mL of 0.9% sterile NaCl solution, then re-centrifuged at 12,000 × *g* for 5 min to collect pellets for DNA extraction. Genomic DNA was extracted using the Bead-Beat Micro AX Gravity kit (A&A Biotechnology, Gdansk, Poland; 106-100-M1) and quantified using a Qubit fluorometer (ThermoFisher Scientific, USA), following the manufacturer’s protocol. Sequencing libraries were prepared using V14 chemistry kits from Oxford Nanopore Technologies: Rapid Barcoding (SQK-RBK114.96) and Native Barcoding sequencing Kit (SQK-NBD114.96), following the standard protocols: RBK_9176_v114_revG_27Nov2022 and NBE_9171_v114_revO_15Sep2022, respectively. The final washing of pooled libraries used Long Fragment Buffer to size-selected for fragments >3 kbp. The libraries were sequenced on a PromethION 2 Solo device for 100 h using R10.4.1 flow cells (Oxford Nanopore Technologies, UK).

Sequencing data were base-called and adapter-trimmed using high-accuracy mode of Guppy basecaller (v6.3.8). *De novo* assembly was conducted with a genome recovery pipeline (https://github.com/yanhui09/ONTrapid), using Flye (v2.9.2) to assemble high-quality reads (*Q* > 10, minimum length >500 bp) into contigs (9). These contigs were polished with two rounds of Racon (v1.5.0) (10) and one round of Medaka (v1.11.1) (11) using model r1041_e82_260bps_hac_v4.1.0. Genome completeness and quality were assessed by BUSCO (v5.5.0) (12), and taxonomic classification was performed using GTDB-Tk (v2.3.0) (13). Default parameters were used except where otherwise noted.

**Fig 1:**
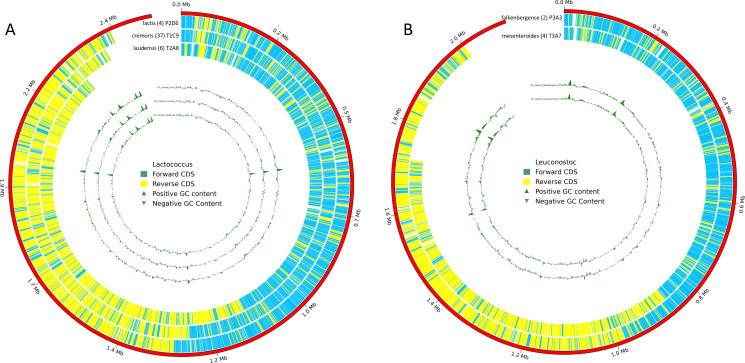
Circular genomic maps of representative strain of *Lactococcus* and *Leuconostoc*. A: GC content (Rings 1–3, respectively), orientation of coding sequences (CDSs, rings 4–6, respectively) and genome size (ring 7) of strain T2A8, T1C9, and P2D6 from *Lactococcus*; B: GC content (Rings 1–2, respectively), orientation of coding sequences (CDSs, rings 3–4, respectively) and genome size (ring 5) of strain T3A7, and P3A3 from *Leuconostoc*.

The dataset and Fig. 1 (function https://github.com/farhadm1990/ONT_helper) display diversity among *Lactococcus* and *Leuconostoc* strains from two starter cultures. The genome sizes range from 2.30 to 2.81 Mbp for *Lactococcus* spp. and from 1.74 to 2.13 Mbp for *Leuconostoc* spp., indicating over 95.2% assembly completeness. Additionally, it reveals uncommon strains *Leuconostoc falkenbergense* and *Lactococcus laudensis*, and a notable absence of *Lactococcus lactis* in Tistrup. These genome analyses enhance our understanding of lactic acid bacteria in dairy fermentation processes.

## Data Availability

Raw sequencing data and the assembled genomes are available at NCBI under the Bioproject accession number PRJNA1073000.

## References

[1] Zheng X, Shi X, Wang B. 2021. A review on the general cheese processing technology, flavor biochemical pathways and the influence of yeasts in cheese. Front Microbiol 12:703284. doi:10.3389/fmicb.2021.703284 34394049 PMC8358398

[2] Montel M-C, Buchin S, Mallet A, Delbes-Paus C, Vuitton DA, Desmasures N, Berthier F. 2014. Traditional cheeses: rich and diverse microbiota with associated benefits. Int J Food Microbiol 177:136–154. doi:10.1016/j.ijfoodmicro.2014.02.019 24642348

[3] Saak CC, Pierce EC, Dinh CB, Portik D, Hall R, Ashby M, Dutton RJ. 2023. Longitudinal, multi-platform metagenomics yields a high-quality genomic catalog and guides an in vitro model for cheese communities. mSystems 8:e0070122. doi:10.1128/msystems.00701-22 36622155 PMC9948695

[4] Terzaghi BE, Sandine WE. 1975. Improved medium for lactic streptococci and their bacteriophages. Appl Microbiol 29:807–813. doi:10.1128/am.29.6.807-813.1975 16350018 PMC187084

[5] Frantzen C, Kleppen HP, Holo H. 2016. Use of M17 and a milk-based medium enables isolation of two distinct and diverse populations of Lactococcus lactis strains from undefined mesophilic starter cultures. Int Dairy J 53:45–50. doi:10.1016/j.idairyj.2015.09.005

[6] Medina R, Katz M, Gonzalez S, Oliver G. 2001. Characterization of the lactic acid bacteria in Ewe’s milk and cheese from Northwest Argentina. J Food Prot 64:559–563. doi:10.4315/0362-028x-64.4.559 11307898

[7] Jain C, Rodriguez-R LM, Phillippy AM, Konstantinidis KT, Aluru S. 2018. High throughput ANI analysis of 90K prokaryotic genomes reveals clear species boundaries. Nat Commun 9:5114. doi:10.1038/s41467-018-07641-9 30504855 PMC6269478

[8] van Mastrigt O, Gallegos Tejeda D, Kristensen MN, Abee T, Smid EJ. 2018. Aroma formation during cheese ripening is best resembled by Lactococcus lactis retentostat cultures. Microb Cell Fact 17:104. doi:10.1186/s12934-018-0950-7 29973201 PMC6030761

[9] Kolmogorov M, Yuan J, Lin Y, Pevzner PA. 2019. Assembly of long, error-prone reads using repeat graphs. Nat Biotechnol 37:540–546. doi:10.1038/s41587-019-0072-8 30936562

[10] Fang L, Wang K. 2022. Polishing high-quality genome assemblies. Nat Methods 19:649–650. doi:10.1038/s41592-022-01515-1 35610477

[11] Lee JY, Kong M, Oh J, Lim J, Chung SH, Kim J-M, Kim J-S, Kim K-H, Yoo J-C, Kwak W. 2021. Comparative evaluation of nanopore polishing tools for microbial genome assembly and polishing strategies for downstream analysis. Sci Rep 11:20740. doi:10.1038/s41598-021-00178-w 34671046 PMC8528807

[12] Waterhouse RM, Seppey M, Simão FA, Manni M, Ioannidis P, Klioutchnikov G, Kriventseva EV, Zdobnov EM. 2018. BUSCO applications from quality assessments to gene prediction and phylogenomics. Mol Biol Evol 35:543–548. doi:10.1093/molbev/msx319 29220515 PMC5850278

[13] Chaumeil P-A, Mussig AJ, Hugenholtz P, Parks DH. 2020. GTDB-TK: a toolkit to classify genomes with the genome taxonomy database. Bioinformatics 36:1925–1927. doi:10.1093/bioinformatics/btz848 PMC7703759 31730192

